# Development of an Early Warning Model for Predicting the Death Risk of Coronavirus Disease 2019 Based on Data Immediately Available on Admission

**DOI:** 10.3389/fmed.2021.699243

**Published:** 2021-08-19

**Authors:** Hai Wang, Haibo Ai, Yunong Fu, Qinglin Li, Ruixia Cui, Xiaohua Ma, Yan-fen Ma, Zi Wang, Tong Liu, Yunxiang Long, Kai Qu, Chang Liu, Jingyao Zhang

**Affiliations:** ^1^Department of Hepatobiliary Surgery, The First Affiliated Hospital of Xi'an Jiaotong University, Xi'an, China; ^2^Rehabilitation Medicine Department, The Third Hospital of Mianyang, Sichuan Mental Health Center, Mianyang, China; ^3^Department of Clinical Laboratory, The First Affiliated Hospital of Xi'an Jiaotong University, Xi'an, China; ^4^Department of Surgical ICU, The First Affiliated Hospital of Xi'an Jiaotong University, Xi'an, China

**Keywords:** COVID-19, early warning, prediction model, death risk, visualization tools

## Abstract

**Introduction:** COVID-19 has overloaded worldwide medical facilities, leaving some potentially high-risk patients trapped in outpatient clinics without sufficient treatment. However, there is still a lack of a simple and effective tool to identify these patients early.

**Methods:** A retrospective cohort study was conducted to develop an early warning model for predicting the death risk of COVID-19. Seventy-five percent of the cases were used to construct the prediction model, and the remaining 25% were used to verify the prediction model based on data immediately available on admission.

**Results:** From March 1, 2020, to April 16, 2020, a total of 4,711 COVID-19 patients were included in our study. The average age was 63.37 ± 16.70 years, of which 1,148 (24.37%) died. Finally, age, SpO2, body temperature (T), and mean arterial pressure (MAP) were selected for constructing the model by univariate analysis, multivariate analysis, and a review of the literature. We used five common methods for constructing the model and finally found that the full model had the best specificity and higher accuracy. The area under the ROC curve (AUC), specificity, sensitivity, and accuracy of full model in train cohort were, respectively, 0.798 (0.779, 0.816), 0.804, 0.656, and 0.768, and in the validation cohort were, respectively, 0.783 (0.751, 0.815), 0.800, 0.616, and 0.755. Visualization tools of the prediction model included a nomogram and an online dynamic nomogram (https://wanghai.shinyapps.io/dynnomapp/).

**Conclusion:** We developed a prediction model that might aid in the early identification of COVID-19 patients with a high probability of mortality on admission. However, further research is required to determine whether this tool can be applied for outpatient or home-based COVID-19 patients.

## Introduction

Since the worldwide COVID-19 epidemic in 2019, up to now (2021/04/03), 129 million people had been infected, and 2.82 million people had died, and the number of confirmed patients with COVID-19 infection was continually growing by hundreds of thousands every day (1), leaving global medical institutions overburdened (2). Because of the substantial growth in COVID-19, several nations are experiencing serious shortages of regular hospital beds and ICU beds (3). As a result, a substantial proportion of COVID-19 patients were trapped in outpatient clinics or at home, unable to receive proper therapy (4); among these there were some patients with a potentially high risk of death. How to early and effectively identify a COVID-19 patient with a high risk of death is a major challenge we face. Although there are more than 100 prediction models about the prognosis of COVID-19 (5, 6), there are relatively few early warning models about the severity of COVID-19. Qing-Lei Gao built an early death risk prediction tool for COVID-19 through machine learning (7, 8). Although the model had high prediction accuracy, the modified model comprised 14 variables, the majority of which were laboratory indicators, making it hard to acquire useful indications immediately on admission. The effect of early warning (7) on admission could not be realized, and because this study did not provide a visual prediction tool, its operability was poor. Furthermore, several researchers investigated other scoring systems such as QSOFA, SOFA, early warning score (EWS), and national early warning Score 2 (NEWS 2) for early warning of the severity of patients with COVID-19. Among them, NEWS2 had a higher warning value for the severity of patients with COVID-19 (9–11). However, these studies about NEWS2 were with minimal sample size, and the score contains eight variables, which made it more difficult to use and affected its clinical application value.

To summarize, the current prediction model or prior illness severity scores were almost all that was required to get laboratory indicators and a large number of items. As a result, completing a COVID-19 severity evaluation and early warning in a timely manner is difficult. More importantly, no matter what prediction model or illness severity scores were used, they were all extremely inconvenient. Therefore, it is necessary to develop a more straightforward prediction tool for predicting the death risk of COVID-19.

## Materials and Methods

### Study Design

A retrospective cohort study.

### Objective

To develop a simple and effective prediction model based on data immediately available on admission to early predict the death risk of COVID-19.

### Setting

Four hospitals in New York City.

### Data Source

The data in this study were shared by Altschul, David and stored in Dryad Database (https://datadryad.org/stash/dataset/10.5061/dryad.7d7wm37sz) (12– 14).

### Diagnosis of COVID-19

SARS-CoV-2 RNA was detected by RT-PCR, and the positive patients were diagnosed as COVID-19 patients.

### Inclusion Criteria

(a) Patients diagnosed as COVID-19 and older than 18 years old; (b) For patients admitted to hospital many times, only the last admission was included for analysis.

### Exclusion Criteria

(a) Although the patient was evaluated in the emergency room, the patient was not admitted to the hospital; (b) Patients who died in the emergency room.

### Participants

From March 1, 2020, to April 16, 2020, patients infected with COVID-19 diagnosed by RT-PCR were collected. The follow-up ended on May 7, 2020, and the follow-up varied from 3 weeks to 80 days. Among them, a total of 4,711 cases confirmed by COVID-19 met the inclusion and exclusion criteria and were included in this study.

### Ethics Statement

New ethics approval was not applicable because the original author had obtained ethical approval when conducting this study. Permission to participate was also not appropriate because our review was a retrospective study of data reuse, and the message of the patients was anonymous.

### Data Immediately Available on Admission Included

**(**a) Demographic data only include age and race, while other relevant data were not provided in the data set, so it could not be included in our study for further analysis; (b) Past medical history included myocardial infarction, congestive heart failure, cerebrovascular disease, diabetes, dementia, and chronic obstructive pulmonary disease (COPD); (c) The vital signs at admission include SpO2, mean artistic pressure (MAP), and body temperature (T). All the above variables were collected on admission.

### Collection of Outcome Indicators

Death-related data were collected through hospital death registration and deaths in the national death registry.

### Selection of Predictor Variables

The following three ways were used to select the variables for the model construction and then construct the corresponding models: (a) All variables that can be obtained immediately on admission were included in the construction and verification of the prediction model; (b) All variables that could be obtained immediately on admission were included in multivariate analysis, and variables with *P*-value < 0.05 were included in the construction and verification of the model; (c) According to the literature review, we further constructed a more concise prediction model.

### Statistical Analysis

(a) Mean ± S.D ($\bar{\text{x}}$ ± s) was used for measurement data, while *n* (%) was used for counting data. (b) Seventy-five percent of the sample size was used to construct the prediction model, and the remaining 25% was used to verify the prediction model. (c) The following methods were used to construct and verify the prediction model, including multiple fractional multivariate models (MFP model), full model, stepwise selected model (stepwise model), bootstrap full (bootstrap resampling 500 times), and bootstrap stepwise (bootstrap resampling 500 Times). (d) The corresponding nomogram was constructed based on the best model described above, and then we used the “DynNom” package to construct a corresponding online dynamic nomogram (15). (e) The missing value of variables included in our study was very few, so there was no special handling of the missing values during model building. Statistical analysis was performed using Empower Stats version 2020 epidemiology software (www.empowerstats.com) and R software.

## Results

### The Clinical Characteristics of Patients

This study comprised 4,711 verified COVID-19 patients who satisfied the inclusion and exclusion criteria. The patients' mean age of the patients was 63.37 ± 16.70 years old, and the races of Black, White, Asian, and Latino were 1,743 (37.00%), 466 (9.89%), 121 (2.57%), and 1,753 (37.21%), respectively, and their SpO2 was 92.89 ± 8.11%, T was 37.31 ± 0.90°C, in which those complicated with myocardial infarction, congestive heart failure, cerebrovascular disease, diabetes, dementia, and COPD were, respectively, 201 (4.27%), 541 (11.48%), 506 (10.74%), 686 (14.56%), 372 (7.90%), and 265 (5.63%), and death cases were 1,148 (24.37%) (See Table 1).

**Table 1:** The clinical characteristics of patients.

**Patient characteristics**	**Mean ± SD/N (%)**
Age, year	63.37 ± 16.70
SpO2, %	92.89 ± 8.19
Mean arterial pressure (MAP), mmHg	85.79 ± 16.81
Temperature, °C	37.31 ± 0.90
**Race**	
Black, *n* (%)	1,743 (37.00%)
White, *n* (%)	466 (9.89%)
Asian, *n* (%)	121 (2.57%)
Latino, *n* (%)	1,753 (37.21%)
Myocardial infarction, *n* (%)	201 (4.27%)
Congestive heart failure, *n* (%)	541 (11.48%)
Cerebrovascular disease, *n* (%)	506 (10.74%)
Diabetes, *n* (%)	686 (14.56%)
Dementia, *n* (%)	372 (7.90%)
COPD, *n* (%)	265 (5.63%)
Death	1,148 (24.37%)

### Univariate Analysis Results

Univariate analysis was performed for the following variables: age, SpO2, MAP, T, black, Asian, White, Latino, myocardial infarction, congestive heart failure, cerebrovascular disease, diabetes, dementia, and COPD. Univariate analysis showed that age, SpO2, MAP, White and COPD were shown to be associated with patient prognosis, with OR values of 1.051 (1.045, 1.056), 0.946 (0.938, 0.954), 0.947 (0.943, 0.952), 1.286 (1.040, 1.591), and1.368 (1.043, 1.794) (See Table 2).

**Table 2:** The results of univariate analysis and multivariate logistic regression analysis.

**Exposure**	**Univariate,** ** OR (95% CI), *P***	**Multivariate,** ** OR (95% CI), *P***
Age, year	1.051 (1.045, 1.056), <0.001	1.052 (1.046, 1.058), <0.001
SpO2, %	0.946 (0.938, 0.954), <0.001	0.954 (0.945, 0.963), <0.001
MAP, mmHg	0.947 (0.943, 0.952), <0.001	0.952 (0.947, 0.957), <0.001
Temperature, °C	1.039 (0.965, 1.120), 0.310	1.109 (1.015, 1.211), 0.022
**Black**, ***n*****(%)**		
No	Reference	Reference
Yes	0.920 (0.801, 1.057), 0.239	0.953 (0.765, 1.188), 0.671
**White**, ***n*****(%)**		
No	Reference	Reference
Yes	1.286 (1.040, 1.591), 0.020	0.849 (0.634, 1.137), 0.272
**Asian**, ***n*****(%)**		
No	Reference	Reference
Yes	1.435 (0.972, 2.120), 0.069	1.788 (1.109, 2.883), 0.017
**Latino**, ***n*****(%)**		
No	Reference	Reference
Yes	0.896 (0.780, 1.029), 0.120	0.848 (0.683, 1.054), 0.137
**Myocardial infarction**, ***n*****(%)**		
No	Reference	Reference
Yes	1.117 (0.810, 1.540), 0.450	1.065 (0.674, 1.683), 0.786
**Congestive heart failure**, ***n*****(%)**		
No	Reference	Reference
Yes	1.219 (0.997, 1.491), 0.053	1.108 (0.844, 1.455), 0.458
**Cerebrovascular disease**, ***n*****(%)**		
No	Reference	Reference
Yes	1.121 (0.908, 1.383), 0.288	0.881 (0.673, 1.152), 0.354
**Diabetes**, ***n*****(%)**		
No	Reference	Reference
Yes	1.008 (0.835, 1.217), 0.936	0.897 (0.684, 1.177), 0.432
**Dementia**, ***n*****(%)**		
No	Reference	Reference
Yes	1.207 (0.952, 1.531), 0.121	1.114 (0.832, 1.492), 0.469
**COPD**, ***n*****(%)**		
No	Reference	Reference
Yes	1.368 (1.043, 1.794), 0.024	1.154 (0.829, 1.605), 0.396

### The Result of Multivariate Logistic Regression Analysis

Multivariate logistic regression analysis was performed for the following variables: age, SpO2, MAP, T, black, Asian, white, Latino, myocardial infarction, congestive heart failure, cerebrovascular disease, diabetes, dementia, and COPD. The multivariate logistic regression analysis revealed that age, SpO2, MAP, T, and Asian were associated with the prognosis of patients with COVID-19, and their OR values were, respectively, 1.052 (1.046, 1.058), 0.954 (0.945, 0.963), 0.952 (0.947, 0.957), 1.109 (1.015, 1.211), and 1.788 (1.109, 2.883) (See Table 2).

### The Construction and Verification of the Prediction Model

**Figure 1:**
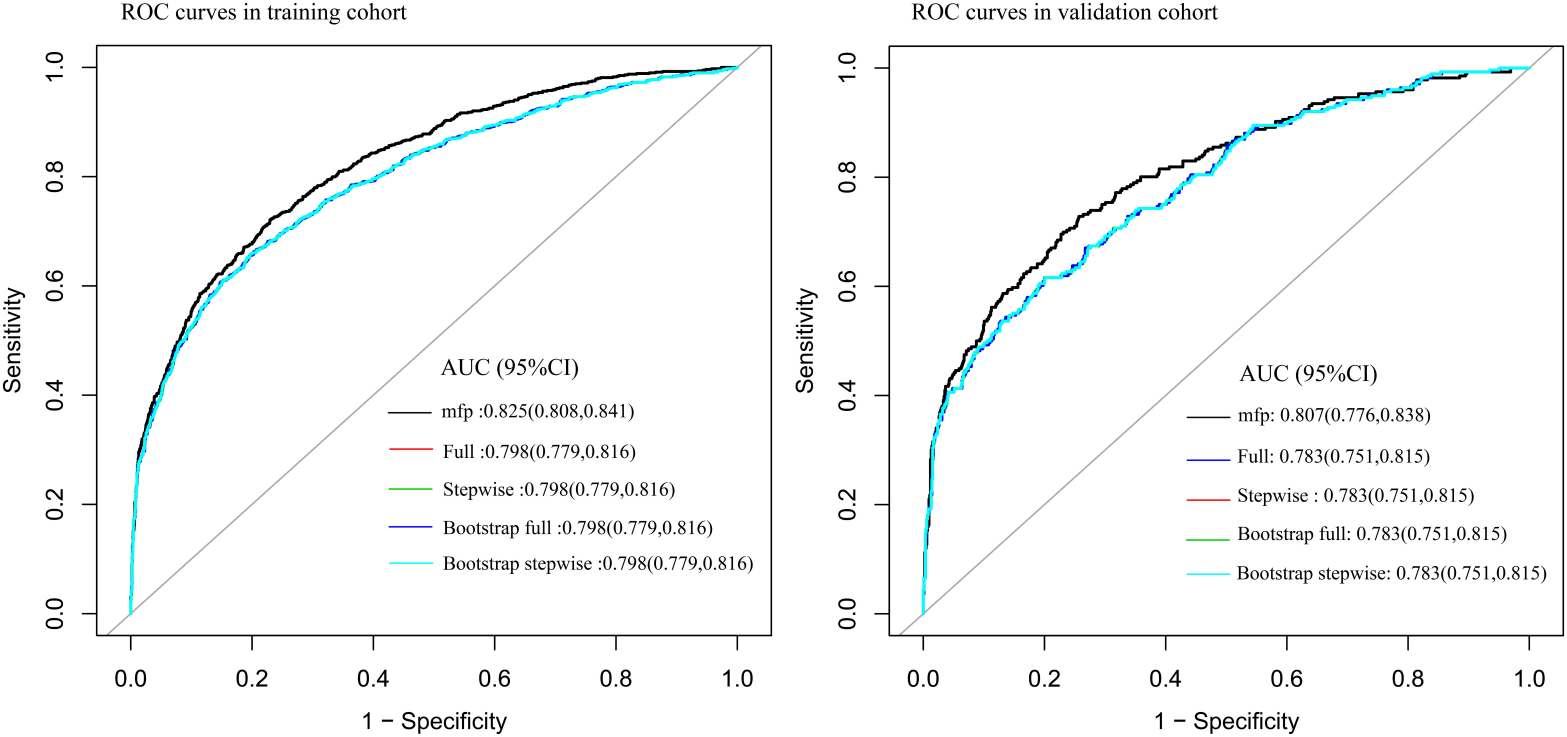
The ROC curve of the predictive model in training cohort and validation cohort.

Seventy-five percent of the sample was used to construct the prediction model: (1) Firstly, age, SpO2, MAP, T, Black, Asian, White, Latino, myocardial infarction, congestive heart failure, cerebrovascular disease, diabetes, dementia, and COPD were all included for constructing the prediction model. The AUC of MFP model, full model, stepwise model, bootstrap full, and bootstrap stepwise were, respectively, 0.828 (0.8115, 0.845), 0.802 (0.784, 0.821), 0.802 (0.783, 0.820), 0.802 (0.784, 0.821), and 0.802 (0.783, 0.820). (2) Secondly, according to the results of multivariate logistic regression analysis, age, SpO2, MAP, T, and Asian were included for constructing the prediction model, and its AUC of MFP model, full model, stepwise model, bootstrap full, and bootstrap stepwise were, respectively, 0.827 (0.811, 0.844), 0.800 (0.782, 0.819), 0.800 (0.782, 0.819), 0.800 (0.782, 0.819), and 0.800 (0.782, 0.819). (3) Finally, age, SpO2, MAP, and T were included for constructing the prediction model, and the AUC of MFP model, full model, stepwise model, bootstrap full, and bootstrap stepwise were, respectively, 0.825 (0.808, 0.841), 0.798 (0.779, 0.816), 0.798 (0.779, 0.816), 0.798 (0.779, 0.816) and 0.798 (0.779, 0.816) (See Table 3; Figure 1.)

**Table 3:** The construction and validation of prediction model.

**Model**	**AUC**	**Specificity**	**Sensitivity**	**Accuracy**
**METHOD 1**				
**Training cohort**				
MFP model	0.828 (0.8115, 0.845)	0.761	0.734	0.755
Full model	0.802 (0.784, 0.821)	0.809	0.658	0.772
Stepwise model	0.802 (0.783, 0.820)	0.789	0.677	0.762
Bootstrap full	0.802 (0.784, 0.821)	0.813	0.652	0.774
Bootstrap stepwise	0.802 (0.783, 0.820)	0.794	0.674	0.765
**Validation cohort**				
MFP model	0.804 (0.774, 0.835)	0.721	0.736	0.724
Full model	0.782 (0.750, 0.814)	0.815	0.590	0.760
Stepwise model	0.780 (0.748, 0.812)	0.864	0.540	0.784
Bootstrap full	0.782 (0.750, 0.814)	0.807	0.598	0.756
Bootstrap stepwise	0.779 (0.747, 0.810)	0.815	0.580	0.757
**METHOD 2**				
**Training cohort**				
MFP model	0.827 (0.811, 0.844)	0.760	0.733	0.753
Full model	0.800 (0.782, 0.819)	0.790	0.673	0.762
Stepwise model	0.800 (0.782, 0.819)	0.790	0.673	0.762
Bootstrap full	0.800 (0.782, 0.819)	0.789	0.674	0.761
Bootstrap stepwise	0.800 (0.782, 0.819)	0.796	0.668	0.765
**Validation cohort**				
MFP model	0.806 (0.776, 0.837)	0.730	0.732	0.731
Full model	0.782 (0.751, 0.814)	0.800	0.612	0.754
Stepwise model	0.782 (0.751, 0.814)	0.800	0.612	0.754
Bootstrap full	0.783 (0.751, 0.814)	0.792	0.620	0.749
Bootstrap stepwise	0.782 (0.750, 0.814)	0.793	0.620	0.750
**METHOD 3**				
**Training cohort**				
MFP model	0.825 (0.808, 0.841)	0.770	0.721	0.759
Full model	0.798 (0.779, 0.816)	0.804	0.656	0.768
Stepwise model	0.798 (0.779, 0.816)	0.804	0.656	0.768
Bootstrap full	0.798 (0.779, 0.816)	0.797	0.663	0.765
Bootstrap stepwise	0.798 (0.779, 0.816)	0.803	0.658	0.768
**Validation cohort**				
MFP model	0.807 (0.776, 0.838)	0.743	0.728	0.740
Full model	0.783 (0.751, 0.815)	0.800	0.616	0.755
Stepwise model	0.783 (0.751, 0.815)	0.800	0.616	0.755
Bootstrap full	0.783 (0.751, 0.815)	0.799	0.616	0.754
Bootstrap stepwise	0.783 (0.751, 0.815)	0.799	0.616	0.754

*The variables of method 1: age, SpO2, MAP, temperature, Black, White, Asian, Latino, myocardial infarction, congestive heart failure, cerebrovascular disease, diabetes, dementia and COPD; the variables of method 2: age, SpO2, MAP, temperature and Asian; the variables of method 3: age, SpO2, MAP and temperature*.

**Figure 2:**
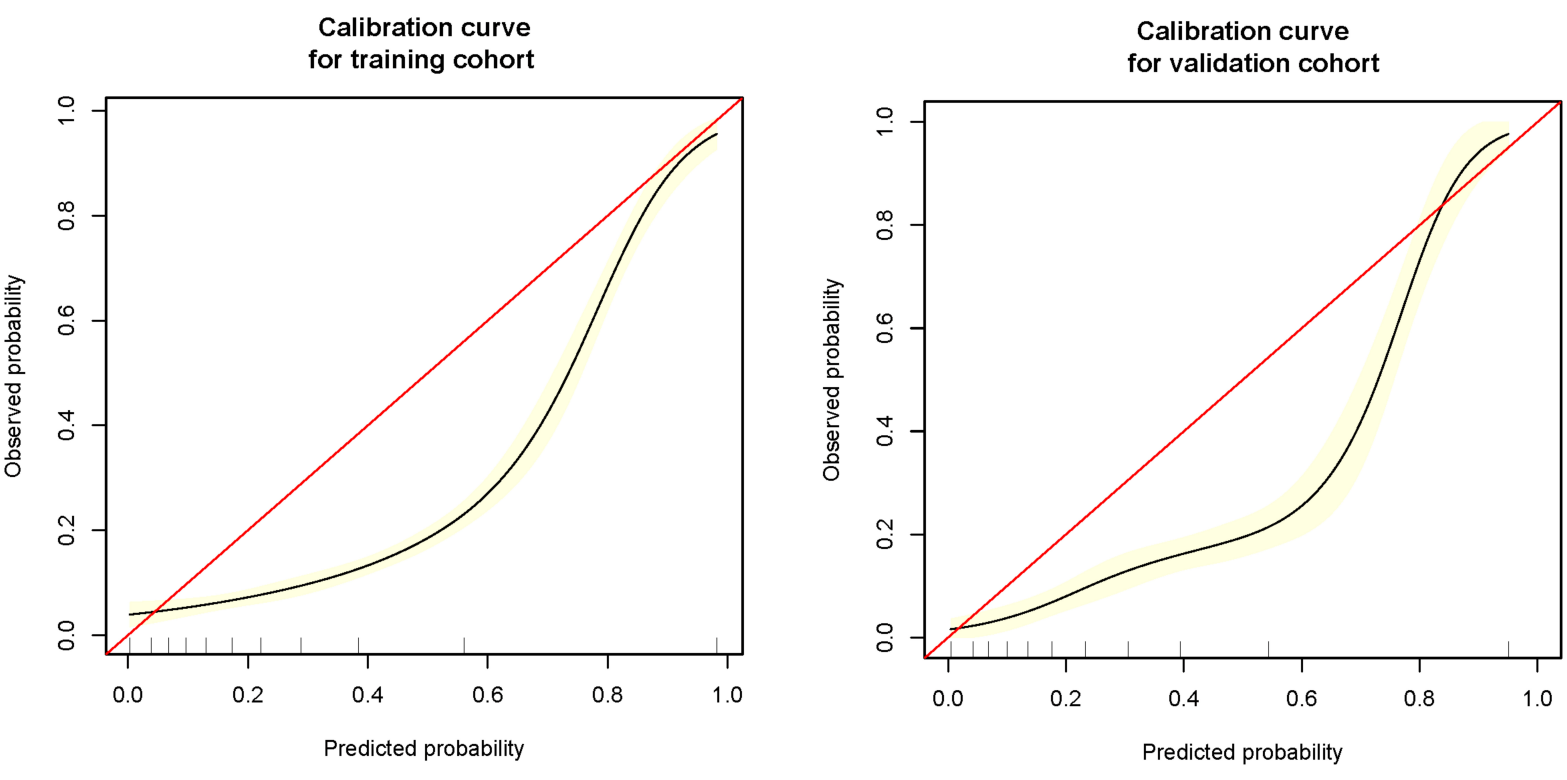
The nomogram of the full model.

The remaining 25% was used to verify the prediction model: (1) Firstly, age, SpO2, MAP, T, Black, Asian, White, Latino, myocardial infarction, congestive heart failure, cerebrovascular disease, diabetes, dementia, and COPD all were included for verifying the prediction model, and its AUC of MFP model, full model, stepwise model, bootstrap full, and bootstrap stepwise were, respectively, 0.804 (0.774, 0.835), 0.782 (0.750, 0.814), 0.780 (0.748, 0.812), 0.782 (0.750, 0.814), and 0.779 (0.747, 0.810). (2) Secondly, age, SpO2, MAP, T, and Asian were included for verifying the prediction model, and its AUC of MFP model, full model, stepwise model, bootstrap full, and bootstrap stepwise were, respectively, 0.806 (0.776, 0.837), 0.782 (0.751, 0.814), 0.782 (0.751, 0.814), 0.783 (0.751, 0.814), and 0.782 (0.750, 0.814). (3) Finally, age, SpO2, MAP, and T were included for verifying the prediction model, and the AUC of MFP model, full model, stepwise model, bootstrap full, and bootstrap stepwise were, respectively, 0.807 (0.776, 0.838), 0.783 (0.751, 0.815), 0.783 (0.751, 0.815), 0.783 (0.751, 0.815), and 0.783 (0.751, 0.815). The calibration curve of the full model for the training cohort and validation cohort showed that predicted probability>observed probability (See Table 3; Figures 1, 2).

### Visualization Tool Construction

**Figure 3:**
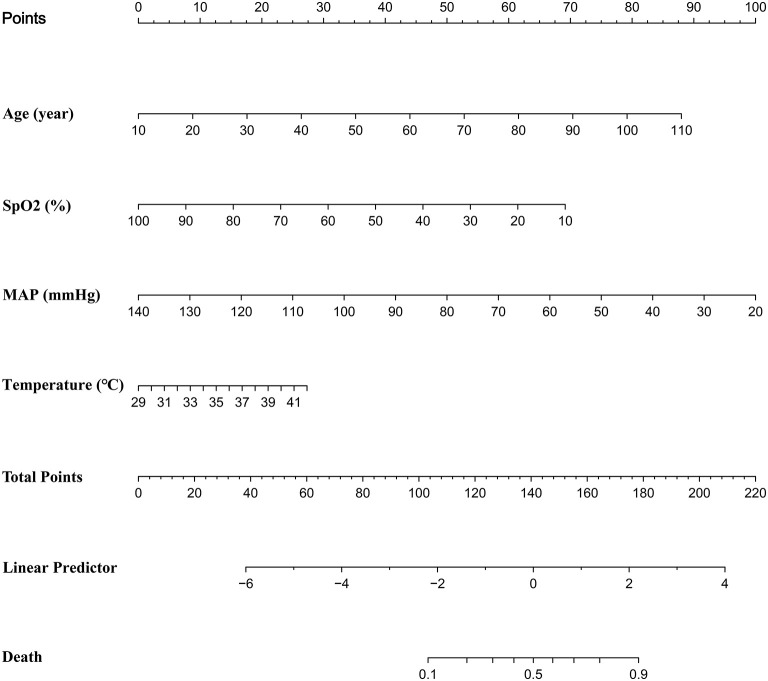
The calibration curve of the full model in training cohort and validation cohort.

We discovered that the prediction model constructed by age, SpO2, MAP, and T had a similar predictive value comparing with the prediction model constructed by other variables. Further, we found that the full model had the highest specificity and similar accuracy, as compared with MFP model, stepwise model, bootstrap full, and bootstrap stepwise. As a result, we chose the Full model as our target prediction model. According to this model, the corresponding nomogram was constructed, and then we used the “DynNom” package to construct a corresponding online dynamic nomogram (https://wanghai.shinyapps.io/dynnomapp/) (See Table 3; Figure 3).

## Discussion

We constructed a prediction model with high predictive value through age, SpO2, MAP, and T, and most important was that the model had high specificity and was simple and easy to be used. All the variables included in the prediction model: age, SpO2, MAP, and T were confirmed to be closely related to the prognosis of COVID-19.

According to some researches, variations in COVID-19 mortality risks across various ethnic groups might be due to economic and cultural differences (16). However, because the data set lacked information on the economy and culture, it was difficult to modify the associated factors to establish whether Asians' death risks were indeed higher than those of other ethnic groups. Furthermore, research has indicated that Asians' death risk is not higher than that of other ethnic groups (17). For the reasons stated above, we did not include Asians as a variable in the prediction model's construction and validation.

A large number of studies had found that age was an independent risk for COVID-19 mortality. In Wuhan, a two-way cohort study involving 548 COVID-19 patients (including 269 severe cases) discovered that the older the patients, the higher the risk of COVID-19 severity and fatality (18). Another study, which included 221 COVID-19 infected individuals, systematically explored the relationship between age and clinical manifestations and prognosis of COVID-19. The study found that elderly patients were more likely to be complicated by bacterial infection, and that the severity of the disease was associated with lower serum albumin levels, higher urea nitrogen levels, higher lactate dehydrogenase levels, and higher inflammatory factors levels, as well as the use of glucocorticoid and ventilator-assisted therapy (19). According to Massimo Volpe's research, the elderly patients had a higher Charlson comorbidity index and higher mortality (20). Wenru Su et al. discovered that SARS-CoV-2 susceptibility gene expression in circulating immune cells increased, as did immune system abnormalities in older individuals (21). To summarize, the elderly patients often had more complications, more likely to be complicated with bacterial infection and hypoproteinemia, immune disorders, and more severity and higher mortality. In this study, the higher the age, the higher the death of patients, consistent with the above studies.

COVID-19 mostly harmed the respiratory system, with acute respiratory distress syndrome being a deadly consequence (22, 23). Ruiguang Zhang et al. found that patients with hypoxemia (SpO2 <90%) had higher levels of IL-6, IL-10, LDH, and C-reactive protein and higher mortality. The above results were consistent with our study.

MAP was one of the indexes reflecting tissue perfusion. A large number of studies showed that MAP on admission was strongly connected to the prognosis of patients. The higher the MAP on admission, the lower the risk of mortality (13, 24).

One of the most prevalent signs of COVID-19 was fever. Dong Chen et al. discovered that around 36% of COVID-19-infected hospitalized patients had a fever, and the greater their body temperature, the worse their prognosis (25). Furthermore, Yongxi Zhang et al. also found that patients with refractory COVID-19 had higher body temperature (26).

To sum up, the variables included in the early warning model: age, SpO2, MAP, and T had been widely confirmed to be closely related to the prognosis of COVID-19, which were also consistent with our research results, so it was reasonable to use the four variables to construct the prediction model.

When compared to previously published prediction models (such as EWS, NEWS2), our prediction model was with relatively low predictive value for the severity of patients with COVID-19. However, these prediction models were with more variables, and meanwhile these variables cannot be obtained in a short time, which made them more difficult to use (9–11). Therefore, these models were not suitable for early warning of COVID-19 severity. However, our model still couldn't instead of these models for subsequent prediction of COVID-19 patients' prognosis. In clinical applications, we might utilize our model for early warning while also combining it with other models to minimize further delays in identifying severely unwell patients.

### The Application Value of This Model

(a) Firstly, we constructed a straightforward prediction tool, besides the traditional nomogram, and we also built a web version of the prediction tool to help doctors or patients predict the death risk of COVID-19 anytime and anywhere. (b) The variables involved in the model of this study could be obtained in a few minutes, without waiting for the laboratory test results for a long time, and could achieve the death risk of COVID-19 at an early stage. (c) The prediction model of our study had high specificity and relatively low sensitivity, which was helpful for doctors to identify those patients with a high risk of death at an early stage, optimize the allocation of medical resources, and alleviate the current shortage of medical resources. (d) The calibration curve showed that the predicted probability was greater than the observed probability in the training cohort and validation cohort. Although our model overestimated the risk of disease (27), our model would be beneficial for physicians to prepare in advance for patients who were likely to develop into severe diseases, and finally improve patients' prognosis. (e) Dynamic Nomogram is a web-based application (28) that integrates measures of AGE, SpO2, T, and MAP. We may use the mouse to choose values of the above four variables and then click the Predict button to calculate the probability of mortality in COVID patients.

### Limitations of Research

(a) Since this study was a retrospective study, further prospective studies would be needed to verify the predictive value of our prediction model. (b) All the cases included in this study were hospitalized patients, which might lead to the limitation of its application population. (c) Our study lacked verification of external validity, the adaptive scope of the model in this study needed to be further verified. Meanwhile, the model in this study needed to be applied cautiously.

## Conclusion

We developed a prediction model that might aid in the early identification of COVID-19 patients with a high probability of mortality on admission. However, further research is required to determine whether this tool can be applied for outpatient or home-based COVID-19 patients.

## Data Availability Statement

The datasets presented in this study can be found in online repositories. The names of the repository/repositories and accession number(s) can be found below: 10.5061/dryad.7d7wm37sz.

## Author Contributions

HW conceived of the study and drafted the manuscript. HA, YF, QL, and RC participated in the statistical analysis. XM, Y-fM, ZW, TL, and YL participated the design of the study. KQ, CL, and JZ participated in its design and coordination and helped to draft the manuscript. All authors contributed to the article and approved the submitted version.

## Conflict of Interest

The authors declare that the research was conducted in the absence of any commercial or financial relationships that could be construed as a potential conflict of interest.

## Publisher's Note

All claims expressed in this article are solely those of the authors and do not necessarily represent those of their affiliated organizations, or those of the publisher, the editors and the reviewers. Any product that may be evaluated in this article, or claim that may be made by its manufacturer, is not guaranteed or endorsed by the publisher.

## Abbreviations

COVID-19: Coronavirus disease 2019
T: body temperature
MAP: mean arterial pressure
AUC: area under the ROC curve
EWS: early warning score
COPD: chronic obstructive pulmonary disease
MFP model: multiple fractional multivariate models
stepwise model: stepwise selected model.

## References

[1] WHO Coronavirus (COVID-19) Dashboard WHO Coronavirus (COVID-19) Dashboard With Vaccination Data. (2021). Available online at: https://covid19.who.int/ (accessed April 03, 2021).

[2] Han E Tan MM Turk E Sridhar D Leung GM Shibuya K. Lessons learnt from easing COVID-19 restrictions: an analysis of countries and regions in asia pacific and Europe. Lancet. (2020) 396:1525–34. 10.1016/S0140-6736(20)32007-9 32979936 PMC7515628

[3] An C Lim H Kim D-W Chang JH Choi YJ Kim SW. Machine learning prediction for mortality of patients diagnosed with COVID-19: a nationwide Korean cohort study. Sci Rep. (2020) 10:18716. 10.1038/s41598-020-75767-2 33127965 PMC7599238

[4] Bchetnia M Girard C Duchaine C Laprise C. The outbreak of the novel severe acute respiratory syndrome coronavirus 2 (SARS-CoV-2): a review of the current global status. J Infect Public Health. (2020) 13:1601–10. 10.1016/j.jiph.2020.07.011 32778421 PMC7402212

[5] Oh B Hwangbo S Jung T Min K Lee C Apio C. Prediction models for clinical severity of COVID-19 patients using multi-center clinical data in Korea. J Med Internet Res. (2021) 23:e25852. 10.2196/25852 33822738 PMC8054775

[6] Domínguez-Olmedo JL Gragera-Martínez Á Mata J Pachón V. Machine learning applied to spanish clinical laboratory data for COVID-19 outcome prediction: model development and validation. J Med Internet Res. (2021) 23:e26211. 10.2196/26211 33793407 PMC8048712

[7] Gao Y Cai G-Y Fang W Li H-Y Wang S-Y Chen L. Machine learning based early warning system enables accurate mortality risk prediction for COVID-19. Nat Commun. (2020) 11:5033. 10.1038/s41467-020-18684-2 33024092 PMC7538910

[8] Bai Z Gong Y Tian X Cao Y Liu W Li J. The rapid assessment and early warning models for COVID-19. Virol Sin. (2020) 35:272–9. 10.1007/s12250-020-00219-0 32239446 PMC7110270

[9] Su Y Ju M-J Xie R-C Yu S-J Zheng J-L Ma G-G. Prognostic accuracy of early warning scores for clinical deterioration in patients with COVID-19. Front Med. (2020) 7:624255. 10.3389/fmed.2020.624255 33598468 PMC7882600

[10] Covino M Sandroni C Santoro M Sabia L Simeoni B Bocci MG. Predicting intensive care unit admission and death for COVID-19 patients in the emergency department using early warning scores. Resuscitation. (2020) 156:84–91. 10.1016/j.resuscitation.2020.08.124 32918985 PMC7480278

[11] Myrstad M Ihle-Hansen H Tveita AA Andersen EL Nygård S Tveit A. National early warning score 2 (NEWS2) on admission predicts severe disease and in-hospital mortality from Covid-19 - a prospective cohort study. Scand J Trauma Resusc Emerg Med. (2020) 28:66. 10.1186/s13049-020-00764-3 32660623 PMC7356106

[12] Eskandar EN Altschul DJ de la Garza Ramos Rafael Cezayirli P Unda SR. Neurologic syndromes predict higher in-hospital mortality in COVID-19. Neurology. (2021) 96:e1527–38. 10.1212/WNL.0000000000011356 33443111 PMC8032378

[13] Altschul DJ Unda SR Benton J de la Garza Ramos Rafael Cezayirli P. A novel severity score to predict inpatient mortality in COVID-19 patients. Sci Rep. (2020) 10:16726. 10.1038/s41598-020-73962-9 33028914 PMC7542454

[14] Altschul David. Neurologic Complications of COVID-19, Dryad, Dataset. (2021). Available online at: 10.5061/dryad.7d7wm37sz (accessed February 01, 2021).

[15] Ding Y Jiang R Chen Y Jing J Yang X Wu X. Comparing the characteristics and predicting the survival of patients with head and neck melanoma versus body melanoma: a population-based study. BMC Cancer. (2021) 21:420. 10.1186/s12885-021-08105-y 33863315 PMC8052690

[16] Townsend MJ Kyle TK Stanford FC. Outcomes of COVID-19: disparities in obesity and by ethnicity/race. Int J Obes. (2020) 44:1807–9. 10.1038/s41366-020-0635-2 32647359 PMC7347050

[17] Webb Hooper M Nápoles AM Pérez-Stable EJ. COVID-19 and racial/ethnic disparities. JAMA. (2020) 323:2466–7. 10.1001/jama.2020.8598 32391864 PMC9310097

[18] Li X Xu S Yu M Wang K Tao Y Zhou Y. Risk factors for severity and mortality in adult COVID-19 inpatients in Wuhan. J Allergy Clin Immunol. (2020) 146:110–8. 10.1016/j.jaci.2020.04.006 32294485 PMC7152876

[19] Liu Y Mao B Liang S Yang J-W Lu H-W Chai Y-H. Association between age and clinical characteristics and outcomes of COVID-19. Eur Respir J. (2020) 55:2001112. 10.1183/13993003.01112-2020 32312864 PMC7173682

[20] Iaccarino G Grassi G Borghi C Ferri C Salvetti M Volpe M. Age and multimorbidity predict death among COVID-19 patients: results of the SARS-RAS study of the italian society of hypertension. Hypertension. (2020) 76:366–72. 10.1161/HYPERTENSIONAHA.120.15324 32564693

[21] Zheng Y Liu X Le W Xie L Li H Wen W. A human circulating immune cell landscape in aging and COVID-19. Protein Cell. (2020) 11:740–70. 10.1007/s13238-020-00762-2 32780218 PMC7417788

[22] Li X Ma X. Acute respiratory failure in COVID-19: is it “typical” ARDS? Crit Care. (2020) 24:198. 10.1186/s13054-020-02911-9 32375845 PMC7202792

[23] Xie J Covassin N Fan Z Singh P Gao W Li G. Association between hypoxemia and mortality in patients with COVID-19. Mayo Clin Proc. (2020) 95:1138–47. 10.1016/j.mayocp.2020.04.006 32376101 PMC7151468

[24] Hajifathalian K Sharaiha RZ Kumar S Krisko T Skaf D Ang B. Development and external validation of a prediction risk model for short-term mortality among hospitalized U.S. COVID-19 patients: a proposal for the COVID-AID risk tool. PLoS ONE. (2020) 15:e0239536. 10.1371/journal.pone.0239536 32997700 PMC7526907

[25] Qiu H Wu J Hong L Luo Y Song Q Chen D. Clinical and epidemiological features of 36 children with coronavirus disease 2019 (COVID-19) in Zhejiang, China: an observational cohort study. Lancet Infect Dis. (2020) 20:689–96. 10.1016/S1473-3099(20)30198-5 32220650 PMC7158906

[26] Chen T Dai Z Mo P Li X Ma Z Song S. Clinical characteristics and outcomes of older patients with coronavirus disease 2019 (COVID-19) in Wuhan, China: a single-centered, retrospective study. J Gerontol A Biol Sci Med Sci. (2020) 75:1788–95. 10.1093/gerona/glaa089 32279081 PMC7184388

[27] Alba AC Agoritsas T Walsh M Hanna S Iorio A Devereaux PJ. Discrimination and calibration of clinical prediction models: users' guides to the medical literature. JAMA. (2017) 318:1377–84. 10.1001/jama.2017.12126 29049590

[28] Chen S Li X Lv H Wen X Ding Q Xue N. Prognostic dynamic nomogram integrated with inflammation-based factors for non-small cell lung cancer patients with chronic hepatitis b viral infection. Int J Biol Sci. (2018) 14:1813–21. 10.7150/ijbs.27260 30443185 PMC6231224

